# Investigating a method for pharmacologic semen collection in alpacas

**DOI:** 10.1590/1984-3143-AR2020-0346

**Published:** 2021-05-28

**Authors:** Anna McAllister, Bernadette Stang, Michelle Anne Kutzler

**Affiliations:** 1 Department of Integrative Biology, Oregon State University, Corvallis, OR, United States; 2 Department of Clinical Sciences, Oregon State University, Corvallis, OR, United States; 3 Department of Animal and Rangeland Sciences, Oregon State University, Corvallis, OR, United States

**Keywords:** camelid, ejaculation, imipramine, sperm, xylazine

## Abstract

While semen evaluation is standard practice prior to a sale or when infertility is suspected in other species, it is rarely done in camelids due to the difficulties involved in collecting a sample. The reproductive physiology of alpacas differs to that of other domestic animals and is still poorly understood. In the stallion, a technique was developed for semen collection that pharmacologically induces ejaculation without copulation (*ex copula*). This study investigates whether semen could be reliably collected by *ex copula* ejaculation in male alpacas. Eleven male Huacaya alpacas were used in this study, and six *ex copula* treatment protocols were evaluated: (1) saline (control); (2) xylazine only (0.1 mg/kg); (3) xylazine only (0.2 mg/kg); (4) imipramine only (1.0 mg/kg); (5) imipramine (1.0 mg/kg) followed 10 minutes later with xylazine (0.1 mg/kg); and (6) imipramine (2.0 mg/kg) followed 10 minutes later with xylazine (0.1 mg/kg). Each treatment protocol was repeated two to five times. Azoospermic samples obtained from *ex copula* ejaculation contained numerous epithelial cells but no sperm. A reliable treatment for pharmacologically inducing ejaculation in alpacas remains to be found.

## Introduction

While semen evaluation is standard practice prior to a sale or when infertility is suspected in other species, it is rarely done in New World camelids due to the difficulties involved in obtaining samples. In camelids, semen collection is complicated by the long duration of copulation and the recumbent position during copulation. Furthermore, ejaculation is a continuous process that occurs throughout copulation lasting 5-50 minutes (San Martin et al., 1968; Lichtenwalner et al., 1996a; Bravo et al., 2002). The ejaculate of camelids is not fractionated, and therefore it has similar semen characteristics throughout the copulatory period (Bravo et al., 2002).

Several methods for semen collection in camelids have been reviewed (Abraham et al., 2016). Each of these methods, however, present unique challenges for semen evaluation. When using intravaginal sacs, the collection device is inserted into the cranial vagina of a receptive female and she is naturally mated (Johnson, 1989; McEvoy et al., 1992). However, in many cases, samples collected using these methods contain no sperm (aspermic) because the male’s penis cannot penetrate the cervix, which triggers ejaculation (Bravo et al., 2000). Furthermore, injury to the female resulting in blood contamination of the ejaculate is also a detriment in using this method of collection (Adams et al., 2009). Additionally, an intravaginal sac often results in reduced copulation time in the male as well as uncooperativeness from the female (Bravo et al., 2000).

For semen collection by electroejaculation, general anesthesia is required (Giuliano et al., 2008; Rodriguez et al., 2012). In addition, semen collected by this method is often contaminated with urine and contains large variations in sperm concentration (61.785-750 x 10^6^/mL) making the analysis of the male as a satisfactory breeder highly variable and potentially unreliable (Sumar, 1985; McEvoy et al., 1992; Fernandez-Baca, 1993; Adams et al., 2009). Additionally, electroejaculation requires equipment that is less accessible and more expensive. Also due to the short duration of ejaculation following electroejaculation, the semen is often less viscous (Lichtenwalner et al., 1996b).

Post-copulatory vaginal aspiration does not require sophisticated instruments and laboratory equipment, making it applicable in any part of the world, but does require a receptive female that has tested negative for contagious diseases (e.g. *Mycoplasma haemolamae*) (Sumar, 1985; Johnson, 1989; McEvoy et al., 1992; Fernandez-Baca, 1993; Bravo et al., 2000; Alarcón et al., 2012; Dascanio, 2014; Viesselmann et al., 2019). This method of semen collection requires that the sample is aspirated from the female genital tract immediately after mating (Tibary, 2003). However, there is often a reduced yield of semen unless the pipette can be passed entirely through the cervix and into the uterus, and it can be unclear if what was aspirated from the vaginal is representative of the ejaculate (Tibary, 2003). Additionally, semen samples obtained using post-copulatory vaginal aspiration are often contaminated with blood from trauma to the female that occurs during copulation or female reproductive tract secretions (Bravo et al., 2000; Alarcón et al., 2012).

Collection of semen by an artificial vagina mounted inside a phantom is considered the “gold standard” for reliable collection of a semen sample for analysis and/or cryopreservation (Lichtenwalner et al., 1996a; Bravo et al., 1997, 2000, 2002). However, males need to be trained to the phantom, which may take several days assuming both an artificial vagina and receptive females are available. Male alpacas are notably less readily trained to the phantom due to the extended copulation time in this species (Adams et al., 2009). Additionally, use of an artificial vagina tends to decrease volume of ejaculate with increasing frequency of use (Lichtenwalner et al., 1996a). There is a need to develop an alternative method for semen collection that can be readily used on the farm prior to a sale of breeding males or as a convenient diagnostic tool for infertility.

In the horse stallion, a technique was developed for semen collection that pharmacologically induces ejaculation without copulation (*ex copula*) (McDonnell et al., 1987; McDonnell, 2001). *Ex copula* ejaculation is induced using a combination of imipramine, a norepinephrine re-uptake inhibitor, and xylazine, an α_2_-agonist, which lowers the ejaculatory threshold centrally and stimulates smooth muscle contractility, respectively. Forty-four of the 64 attempts to induce ejaculation (from 8 stallions) resulted in semen collection following treatment (McDonnell, 2001). Stallions that ejaculated extruded their penis for the duration of copulation. During ejaculation the urethralis and bulbospongiosus muscles contract resulting in pulsation and release of semen. This method allows a semen sample to be obtained from stallions not trained to an artificial vagina, too fractious to be handled or too injured or debilitated to mount a phantom (Turner et al., 1995).

Preliminary work in this laboratory using similar dosages as reported in stallions has suggested that imipramine (1.0 mg/kg IV) followed in 10 minutes by xylazine (0.1 mg/kg IV) can be useful for the induction of *ex copula* ejaculation in llamas (Kutzler et al., 2005). This protocol resulted in ejaculates that contained sperm (< 1 million/mL) 30% of the time without contamination from blood or urine (Kutzler et al., 2005). Extrusion of the penis from the prepuce was not noted irrespective of ejaculation. The goal of the current study was to optimize imipramine and xylazine dosages for reliable pharmacologic ejaculation in alpacas.

## Methods

Eleven intact, adult (6.5 ± 2.6 years) male Huacaya alpacas were used in this study. Males were completely shorn prior to investigations to provide the most accurate determination of body weight for dosage approximations (75.3 ± 12.2 kg). Animals were housed in compatible groups in order to prevent isolation stress. Grass hay and water were offered ad libitum. All studies were approved by the Oregon State University Institutional Animal Care and Use Committee (protocol #3664).

**Figure 1:**
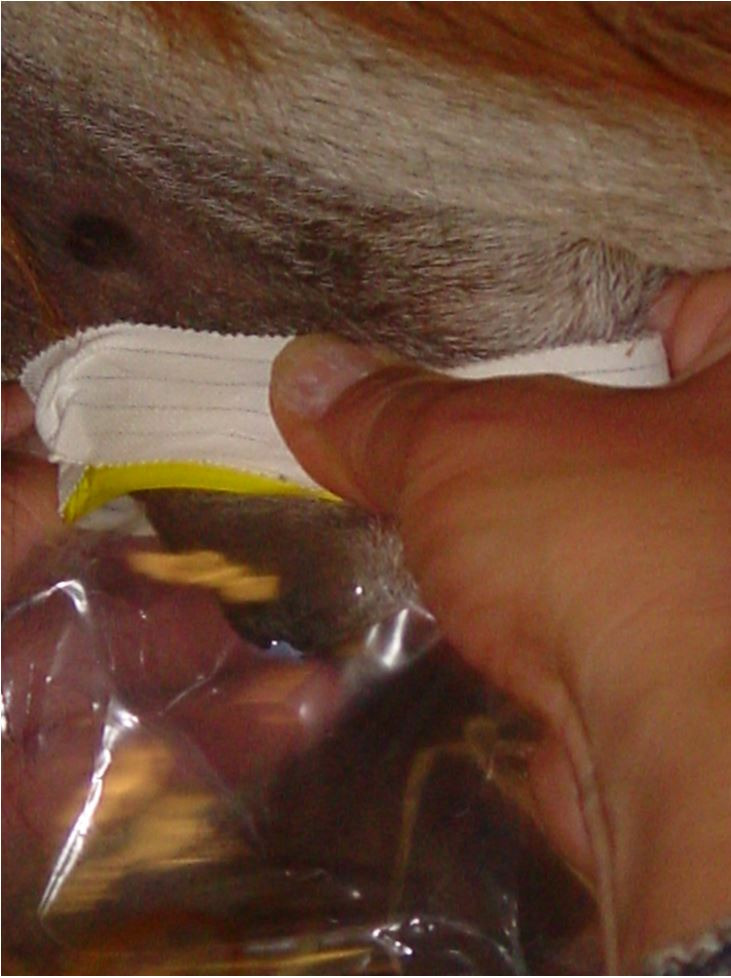
Ejaculates were collected into a Whirl-Pak® bag (Enasco, Fort Atkinson, WI) secured over the preputial opening with tape.

Right jugular intravenous catheters were placed to facilitate daily imipramine and xylazine administration. Imipramine HCL was compounded at a concentration of 25 mg/mL (Pet Pharmacy, NW; Corvallis OR). Prior to each treatment, a Whirlpak® bag was taped over the preputial opening and removed 10-30 minutes following each treatment (Figure 1). Six treatment protocols were evaluated: (1) saline (control), (2) xylazine only (0.1 mg/kg), (3) xylazine only (0.2 mg/kg), (4) imipramine only (1.0 mg/kg), (5) imipramine (1.0 mg/kg) followed 10 minutes with xylazine (0.1 mg/kg), and (6) imipramine (2.0 mg/kg) followed 10 minutes with xylazine (0.1 mg/kg). The order in which each treatment was administered to all eleven males was randomly assigned. Each treatment protocol was replicated two to five times and 24-hours passed between each treatment. The objective of each treatment was to determine a standard dosage for reliable pharmacologic ejaculation in alpacas. Ejaculate samples were placed on pre-warmed glass slides and evaluated with and without stain (Diff-Kwik®, Medion Diagnostics Ag, Duedingen, Switzerland) using light microscopy at 100x and 400x magnification for the presence of spermatozoa. Results were recorded for volume, clarity, color, viscosity, presence of sperm, and presence of other cell types. Descriptive results for pharmacological ejaculation protocols were reported as percentages.

## Results

### Pharmacologic semen collection

**Figure 2:**
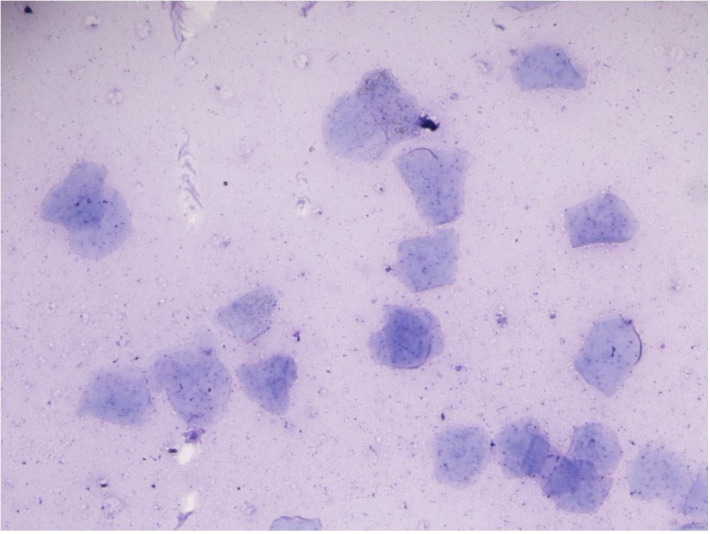
Ejaculate stained with Diff-Kwik® and examined microscopically at 100x magnification showing keratinized epithelial cells in proteinaceous background.

**Figure 3:**
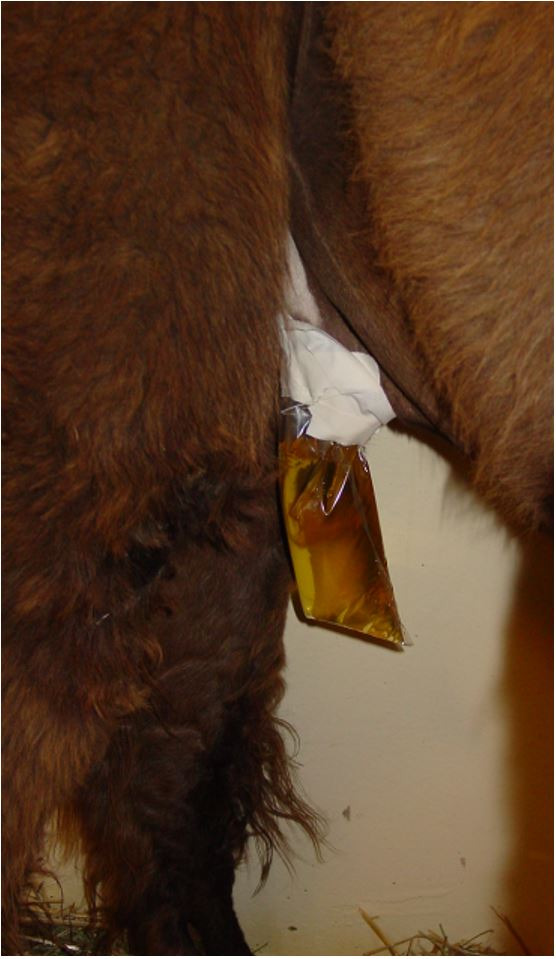
Urine leakage occurred occasionally in response to pharmacologic semen collection.

**Figure 4:**
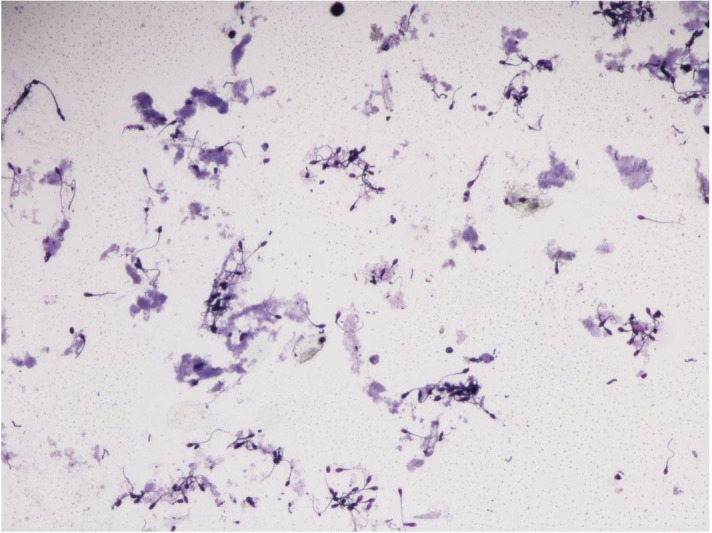
Urine sediment sample from an alpaca following pharmacologic ejaculation. Sample was stained with Diff-Kwik® and examined microscopically at 100x magnification. Few spermatozoa were present in 33.3% (4/12) of urine sediment samples.

Responses to pharmacologic semen collection varied by treatment and individual (Table 1). Azoospermic ejaculates were low volume (<50 µL to 1000 µL), non-viscous, clear fluid samples, presumably from the prostate or bulbourethral glands. Azoospermic ejaculates occurred in 45.5% (5/11) of males following treatment with imipramine only given at the 1 mg/kg dosage. However, this was an inconsistent finding. None of the azoospermic ejaculates contained sperm, but numerous epithelial cells were present in a proteinaceous background (Figure 2). Urine leakage occurred occasionally in response to pharmacologic semen collection (Figure 3). If urine leakage occurred, the entire sample containing urine was centrifuged, and the sediment was stained with Diff-Kwik® and examined microscopically at 100X magnification. Spermatozoa were present in 33.3% (4/12) of urine samples (Figure 4). Two males (18%) did not have any response (ejaculation or urination) to any treatment.

**Table 1:** Responses to pharmacologic ejaculation protocols in eleven alpacas.

**Male ID**	**Saline**		**Xylazine (0.1 mg/kg)**		**Xylazine (0.2 mg/kg)**		**Imipramine (1 mg/kg)**		**Imipramine (1 mg/kg) Xylazine (0.1 mg/kg)**					**Imipramine (2 mg/kg) Xylazine** **(0.1 mg/kg)**		
1	N	N	N	N	N	N	N	N	N	N	N	N	N	N	N	N
2	N	N	N	N	N	N	N	N	N	N	N	N	N	N	N	N
3	N	N	N	N	N	N	E_A_	E_A_	N	N	E_A_	E_A_	E_A_	N	U_A_	E_A_
4	N	N	N	N	N	N	N	N	E_A_	N	N	U_A_	N	N	N	N
5	N	N	N	N	N	E_A_	E_A_	N	N	U_S_	U_S_	N	N	E_A_	N	N
6	E_A_	-	E_A_	N	E_A_	N	N	N	E_A_	E_A_	N	N	-	N	N	E_A_
7	N	N	N	N	N	U_S_	E_A_	N	N	U_A_	U_A_	U_S_	E_A_	N	E_A_	N
8	N	N	N	N	N	N	E_A_	E_A_	N	N	N	U_A_	U_A_	N	N	N
9	N	N	E_A_	N	N	N	N	N	N	N	N	E_A_	N	N	N	N
10	N	N	N	N	N	E_A_	U_A_	N	N	N	N	N	U_A_	N	N	N
11	N	N	N	N	N	N	E_A_	N	N	N	N	N	N	N	N	N

E_A_: Ejaculate with no sperm present; E_S_: Ejaculate with sperm present; N: No ejaculate or urine leakage following treatment; U_A_: Urine with no sperm present; U_S_: Urine with sperm present.

## Discussion

The mechanisms through which xylazine and imipramine act in ejaculation are not fully understood. Ejaculation consists of two physiologic responses: emission and expulsion (Solomon et al., 1997). Emission primarily involves α1-adrenergic stimulation while ejaculation is mediated primarily by α2-adrenergic stimulation (Solomon et al., 1997; Yonezawa et al., 2005). Even though xylazine is primarily an α2-agonist, it is believed to have an effect on both pathways to induce ejaculation (Johnston and DeLuca, 1998). Imipramine is a tricyclic antidepressant that can induce emission and expulsion in men (Breier et al., 1984) and in stallions (McDonnell et al., 1987). Imipramine potentiates the actions of biogenic amines (e.g. norepinephrine) by blocking the re-uptake of amines at nerve terminals (Axelrod et al., 1961). *In vitro* studies show that imipramine stimulates smooth muscle contraction through the vas deferens and ampulla in bulls and has been recommended for administration at 0.1-0.2 mg/kg prior to electroejaculation (Cordel et al., 2001).

The hypothesis of the current study was that semen could be reliably collected by *ex copula* ejaculation in alpacas. This was unsupported in our findings as reliable pharmacologic ejaculation was not achieved. None (0/24) of the pharmacologic induced ejaculates yielded sperm. Imipramine was found to be safe when given at an intravenous dose of 1-2 mg/kg in alpacas. Similar observations were made in llamas during a previous study (Kutzler et al., 2005). However, over-sedation was a common problem in the current study. Imipramine appears to potentiate the sedative effects of xylazine, but its effects are very animal dependent. Over-sedation occurred at both the 1.0 mg/kg and 2.0 mg/kg imipramine dosages followed by a 0.1 mg/kg dose of xylazine in alpacas 10.9% (6/55) and 12.12% (4/33) of the time respectively. Difficulties collecting semen samples from animals after pharmacologic treatment was likely due to over-sedation as optimized levels for pharmacological induction of ejaculation could not be accomplished during this study. In addition, it is not known if the ejaculation pattern in this species could have influenced the results. However, preliminary research in llamas (also continuous ejaculators) demonstrated that induction of *ex copula* ejaculation is possible as 30% of the 29 samples taken contained sperm in this study (Kutzler et al., 2005).

Semen collection through pharmacologic ejaculation was also hindered by stress due to the animals’ lack of familiarity of human contact and training to the halter. The comfort of the animals with humans at the time of treatment has a considerable effect on results and cannot be overemphasized. Animals under stress are less likely to ejaculate (Grønli et al., 2005), which may be the reason that many of the attempts to collect these animals were unsuccessful. Additionally, 27.3% (3/11) of the males had not been previously used for breeding and therefore breeding histories were not available for all of the animals. Further study using the same methods of collection may be more successful with animals that have been better familiarized with human contact and if breeding history was available for all males prior to the investigation. In addition, further study combining pharmacologic stimulus with the use of an artificial vagina or phantom may enhance ejaculation and improve semen collection from alpacas. Alternatively, using a different *ex copula* protocol (e.g. detomidine with oxytocin) may be effective, as demonstrated in stallions when imipramine and xylazine was not effective (Cavalero et al., 2019). It is possible that using a different *ex copula* protocol which results in the extrusion of the penis from the prepuce may be more likely to result in ejaculation. It is important to consider that methods to pharmacologically induce ejaculation in equids may not work in camelids which have a continuous pattern of ejaculation during the process of mating (Bravo et al., 2002).

## Conclusion

Efforts to optimize the dosage required to pharmacologically induce ejaculation in alpacas were unsuccessful as none of the tested dosages resulted in reliable semen collection. Over-sedation and urine leakage were common responses to the pharmacological treatments.

## References

[B001] Abraham MC, Puhakka J, Ruete A, Al-Essawe EM, de Verdier K, Morrell JM, Bage R (2016). Testicular length as an indicator of the onset of sperm production in alpacas under Swedish conditions. Acta Vet Scand.

[B002] Adams GP, Ratto MH, Collins CW, Bergfelt DR (2009). Artificial insemination in South American camelids and wild equids. Theriogenology.

[B003] Alarcón VB, Garcia WV, Bravo PW (2012). Artificial insemination of alpacas with semen collection by vaginal aspiration and by artificial vagina. Rev Investig Vet Peru.

[B004] Axelrod J, Whitby LG, Hertting G (1961). Effect of psychotropic drugs on the uptake of H3-norepinephrine by tissues. Science.

[B005] Bravo PW, Flores U, Garnica J, Ordoñez C (1997). Collection of semen and artificial insemination of alpacas. Theriogenology.

[B006] Bravo PW, Moscoso R, Alarcon V, Ordoñez C (2002). Ejaculator process and related semen characteristics. Arch Androl.

[B007] Bravo PW, Skidmore JA, Zhao XX (2000). Reproductive aspects and storage of semen in Camelidae. Anim Reprod Sci.

[B008] Breier A, Ginsberg EM, Charney DS (1984). Seminal emission induced by tricyclic antidepressant. Am J Psychiatry.

[B009] Cavalero TMS, Papa FM, Schmith RA, Scheeren VFC, Canuto LEF, Gobato MLM, Rodrigues LT, Freitas-Dell’aqua CP (2019). Protocols using detomidine and oxytocin induce ex copula ejaculation in stallions. Theriogenology.

[B010] Cordel C, Swan GE, Mulders MSG, Bertschinger HJ (2001). Pharmacokinetics of intravenous imipramine hydrochloride in cattle. J Vet Pharmacol Ther.

[B011] Dascanio J, Cebra C, Anderson D, Tibary A, Van Saun R, Johnson L (2014). Breeding soundness examination of the llama and alpaca.. llama and alpaca care: medicine, surgery, reproduction and herd health..

[B012] Fernandez-Baca S (1993). Manipulation of reproductive functions in male and female New World camelids. Anim Reprod Sci.

[B013] Giuliano S, Director A, Gambarotta M, Trasorras V, Miragaya M (2008). Collection method, season and individual variation on seminal characteristics in the llama (Lama glama). Anim Reprod Sci.

[B014] Grønli J, Murison R, Fiske E, Bjorvatn B, Sørensen E, Portas CM, Ursin R (2005). Effects of chronic mild stress on sexual behavior, locomotor activity and consumption of sucrose and saccharine solutions. Physiol Behav.

[B015] Johnson LW (1989). Llama reproduction. Vet Clin North Am Food Anim Pract.

[B016] Johnston PF, DeLuca JL (1998). Chemical ejaculation of stallions after the administration of oral imipramine followed by intravenous xylazine..

[B017] Kutzler MA, Stang BV, Hoepp N (2005). Preliminary studies on the use of imipramine and xylazine to induce ejaculation in llamas..

[B018] Lichtenwalner AB, Woods GL, Weber JA (1996). Ejaculatory pattern of llamas during copulation. Theriogenology.

[B019] Lichtenwalner AB, Woods GL, Weber JA (1996). Seminal collection, seminal characteristics and pattern of ejaculation in llamas. Theriogenology.

[B020] McDonnell SM, Garcia MC, Kenney RM, Van Arsdalen KN (1987). Imipramine-induced erection, masturbation, and ejaculation in male horses. Pharmacol Biochem Behav.

[B021] McDonnell SM (2001). Oral imipramine and intravenous xylazine for pharmacologically-induced ex copula ejaculation in stallions. Anim Reprod Sci.

[B022] McEvoy TG, Kyle CE, Young P, Adam CL, Bourke DA (1992). Aspects of artificial breeding and establishment of pregnancy in South American camelids..

[B023] Rodriguez J, Huanca W, Ramos M, Vasquez M, Espinoza J (2012). Biophysical and biochemical characteristics of alpaca semen after collection by electroejaculation. Reprod Fertil Dev.

[B024] San-Martin M, Copaira M, Zuniga J, Rodreguez R, Bustinza G, Acosta L (1968). Aspects of reproduction in the alpaca. J Reprod Fertil.

[B025] Solomon HM, Wier PJ, Ippolito DL, Toscano TV (1997). Effect of prazosin on sperm transport in male rats. Reprod Toxicol.

[B026] Sumar J, Land RB, Robinson DW (1985). Reproductive physiology in South American camelids.. Genetics of reproduction in sheep..

[B027] Tibary A, Hoffman E (2003). Male reproduction.. The complete alpaca book..

[B028] Turner RM, McDonnell SM, Hawkins JF (1995). Use of pharmacologically induced ejaculation to obtain semen from a stallion with a fractured radius. J Am Vet Med Assoc.

[B029] Viesselmann LC, Videla R, Schaefer J, Chapman A, Wyrosdick H, Schaefer DMW (2019). Mycoplasma haemolamae and intestinal parasite relationships with erythrocyte variables in clinically healthy alpacas and llamas. J Vet Intern Med.

[B030] Yonezawa A, Yoshizumi M, Ebiko M, Amano T, Kimura Y, Sakurada S (2005). Long-lasting effects of yohimbine on the ejaculatory function in male dogs. Biomed Res.

